# Supplementary material for: The Reduced Longitudinal Growth Induced by Overexpression of pPLAIIIγ Is Regulated by Genes Encoding Microtubule-Associated Proteins
Source: Plants (Basel). 2021 Nov 28;10(12):2615. doi: 10.3390/plants10122615 (PMC8706840; doi:10.3390/plants10122615)
Supplement: Supplementary file 1 [file plants-10-02615-s001.zip › plants-1446495-supplementary/plants-1446495-figure S1.pdf]

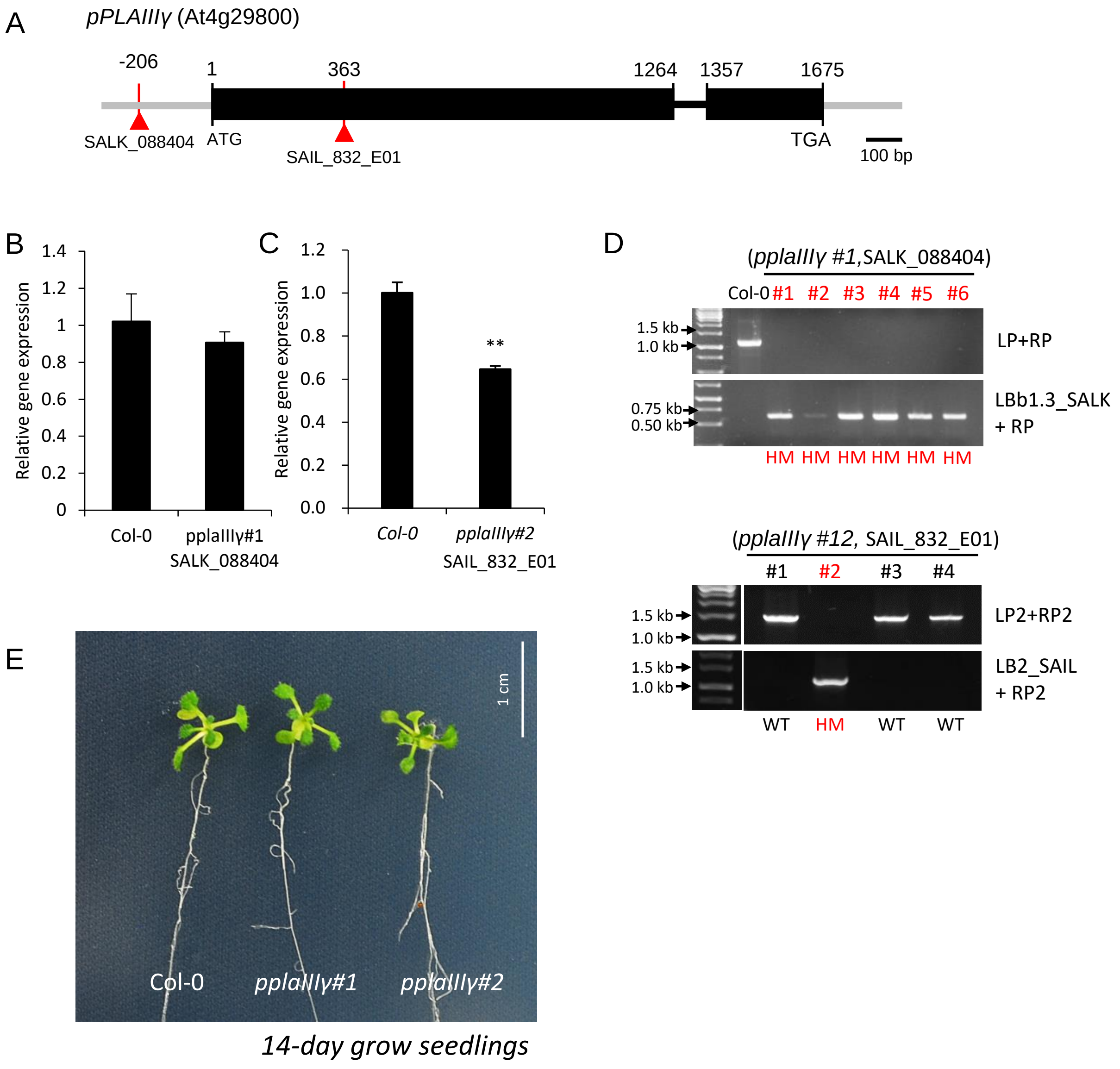

Figure S1. Analysis of T-DNA insertion lines in *pPLAIIIγ* gene. (A) Genomic structure of *pPLAIIIγ* (At4g29800) and the T-DNA insertions is marked with red-filled triangle. Black boxes: exon, black line: intron, and grey lines: UTR. (B and C) Expression level of *pPLAIIIγ* in Col-0 and two *pplaIIIγ* T-DNA mutants. (B) *pplaIIIγ*#1: SALK_088404. (C) *pplaIIIγ*#2: SAIL_832_E01. Data represent the average ± SE from three independent replicates at $P < 0.05$ (*) and $P < 0.01$ (**), respectively. (D) Genotyping PCR of the T-DNA insertion among independent *pplaIIIγ* mutants. HM: homozygous T-DNA insertion, WT: wild type. (E) No phenotypic difference was observed in *pplaIIIγ* mutant lines. Scale bar = 1 cm.
