# Supplementary material for: The Reduced Longitudinal Growth Induced by Overexpression of pPLAIIIγ Is Regulated by Genes Encoding Microtubule-Associated Proteins
Source: Plants (Basel). 2021 Nov 28;10(12):2615. doi: 10.3390/plants10122615 (PMC8706840; doi:10.3390/plants10122615)
Supplement: Supplementary file 1 [file plants-10-02615-s001.zip › plants-1446495-supplementary/Table S1.pdf]

Table S1. List of DNA primers used in this study for confirmation of gene insertion and polymerase chain reaction

Gene	Accession No.	Annotation	Primers used (5' - 3')
pPLAIIIγ	At4g29800	pPLAIIIγ-F (qRT)	GTC CAA CGT TAT CAA CGG CTC GAT
		pPLAIIIγ-R (qRT)	TCC TCC ACT TCC GCT CCC TGA TCT
		pPLAIIIγ-KpnI-F (Cloning)	TC GGT ACC GTC TAA AAG CTA ACG ATT
		pPLAIIIγ-AvrII-R (Cloning)	GG CCT AGG TCT ATC TTT AGA TAT GAG
		pPLAIIIγ-LP (SALK_088404)	TATCATCGCAGCGAATACTCC
		pPLAIIIγ-RP (SALK_088404)	TTGGAATCCATTTGGGAAAAG
		pPLAIIIγ-LP2 (SAIL_832_E01)	TATCATCGCAGCGAATACTCC
		pPLAIIIγ-RP2 (SAIL_832_E01)	TTGGAATCCATTTGGGAAAAG
SALK T-DNA	-	LBb1.3_SALK	ATT TTG CCG ATT TCG GAA C
SAIL T-DNA	-	LB2_SAIL	GCT TCC TAT TAT ATC TTC CCA AAT TAC CAA TAC A
Actin	At5g09810	Atactin-2F	GTG TGT CTT GTC TTA TCT GGT TCG
		Atactin-2R	AAT AGC TGC ATT GTC ACC CGA TAC T
MAP18	At5g44610	MAP18-F	AAG CCA GCT GTG GAA GA
		MAP18-R	TTC GGG AGC CTT AGT
MAP20	At5g37478	MAP20-F	CAA GGC AGC CAC GAC TAA GA
		MAP20-R	TGC ACG TTT CAC TGC TCT CT
MAP65-1	At5g55230	MAP65-1-F	ACA CAG CAT GTC CTT TGC CT
		MAP65-1-R	ACG CCG GTT ATG TGT TCC AT
MAP70-1	At1g68060	MAP70-1-F	CCG AAG TGG GTT AAC AAG GA
		MAP70-1-R	CAC ACC AGC CTA TAA CGG TTT
MAP70-5	At4g17220	MAP70-5-F	CTG TTT CCG AGA GAA CCG CT
		MAP70-5-R	TCT TTG AGC CAC CAC CAC TG
PLDα1	At3g15730	PLDα1-F	TCT CTG CTT TGC TGC TGT TGT AGC
		PLDα1-R	CAC AAA GCT ACA TTC TCT CAC CAC GTC
PLDδ	At4g35790	PLDδ-F	TGC CGG CGA TTG GGT TAG ATA
		PLDδ-R	GAG CAA TTC CCC AAG CGT CAT A
PLDζ1	At3g16785	PLDζ1-F	TGG ATG GCA ACC GCA AAG ACA A

		PLDζ1-R	ATC GTT GTG TGT CCC AGC TTC T
PLDζ2	At3g05630	PLDζ2-F	TTT GAG GAC GGT CCA ATT GCC A
		PLDζ2-R	ACA ACA CCG ATC TCA GAG TCT CGT
ACS4	At2g22810	ACS4-F	GGT TGG GAA GAG TAC GAG AAG
		ACS4-R	GCT GCG TCT GTG TTT TGT G
ACS5	At5g65800	ACS5-F	TCA CGA ATC CAT CTA ACC CAC
		ACS5-R	CCC GAA CAT AGT GCC TGA ATA G
ACS11	At4g08040	ACS11-F	GGT TTG TTC TGT TGG GTT GAC
		ACS11-R	AAT GAC ACG ATG AGC CTG G
ACO1	At2g19590	ACO1-F	TGT CAG ATC CCA AAC ATT TCA G
		ACO1-R	GGG TAT TTA GCC ACT TTT GTT CC
ACO2	At1g62380	ACO2-F	CGG GAA GTA TAA GAG TGT GCT G
		ACO2-R	GGG TAC TCG GAA TCT TTC TCG
ACO4	At1g05010	ACO4-F	TTT CTA CCT CAA GCA CCT TCC
		ACO4-R	CGG CGA AGT CTT TCA TTA ACG
